# Polyamines directly promote antizyme-mediated degradation of ornithine decarboxylase by the proteasome

**DOI:** 10.15698/mic2015.06.206

**Published:** 2015-05-20

**Authors:** R. R. Beenukumar, Daniela Gödderz, R. Palanimurugan, R. J. Dohmen

**Affiliations:** 1 Institute for Genetics, University of Cologne, Biocenter, Zülpicher Str. 47a, D-50674 Cologne, Germany.; 2 Present address: Karolinska Institute, Department for Cell- and Molecular Biology, Von Eulers väg 3, 171 77 Stockholm.; 3 Present address: Center for Cellular and Molecular Biology (CCMB), Uppal Road, Hyderabad 500007, India.

**Keywords:** antizyme, ODC, polyamines, proteasome, ubiquitin

## Abstract

Ornithine decarboxylase (ODC), a ubiquitin-independent substrate of the proteasome, is a homodimeric protein with a rate-limiting function in polyamine biosynthesis. Polyamines regulate ODC levels by a feedback mechanism mediated by ODC antizyme (OAZ). Higher cellular polyamine levels trigger the synthesis of OAZ and also inhibit its ubiquitin-dependent proteasomal degradation. OAZ binds ODC monomers and targets them to the proteasome. Here, we report that polyamines, aside from their role in the control of OAZ synthesis and stability, directly enhance OAZ-mediated ODC degradation by the proteasome. Using a stable mutant of OAZ, we show that polyamines promote ODC degradation in *Saccharomyces cerevisia*e cells even when OAZ levels are not changed. Furthermore, polyamines stimulated the *in vitro* degradation of ODC by the proteasome in a reconstituted system using purified components. In these assays, spermine shows a greater effect than spermidine. By contrast, polyamines do not have any stimulatory effect on the degradation of ubiquitin-dependent substrates.

## INTRODUCTION

The ubiquitin-proteasome system, with the 26S proteasome as its central player, provides a key regulatory protein degradation mechanism in eukaryotes 1. The 26S proteasome is a ~2.6 MDa multi-subunit protease that utilizes ATP for substrate degradation. It consists of two sub-complexes, the barrel-shaped catalytic core particle (CP) and the 19S regulatory particle (RP) 2. The CP is composed of four heptameric rings, two outer α rings and two inner β rings. The latter harbor the proteolytic active sites residing in the β1, β2 and β5 subunits 3. Using biochemical experiments, the RP was characterized to consist of two sub-complexes, the base with its hexameric ATPase ring and ubiquitin receptor subunits (Rpn10 and Rpn13), and the lid including the Rpn11 deubiquitylating activitiy 4. Targeting of substrates to the proteasome is mainly achieved by polyubiquitin-tagging, which leads to recognition by intrinsic ubiquitin receptors of the proteasome or by proteasome-associated shuttling factors 1*.* Recent CryoEM studies have shed light on the details of ubiquitin-dependent substrate recognition and engagement by the proteasome 5,6,7,8*.* Upon substrate recognition through polyubiquitin binding by the receptor, an unstructured region of the substrate gets threaded through the ATPase pore thereby engaging the proteasome. Translocation of the substrate leads to a conformational change that promotes removal of the polyubiquitin chain from the substrate by Rpn11. Subsequently, the rest of the substrate is translocated into the CP 9*.*

Interestingly, certain substrates of the proteasome do not require ubiquitylation for their recognition and degradation. The mechanism of ubiquitin-independent proteasomal targeting still remains unclear. It has been speculated that presence of an unstructured domain in these proteins is sufficient for proteasome association 10*.* The mammalian thymidylate synthase, yeast Rpn4, and Ornithine decarboxylase (ODC) are some of the best-studied ubiquitin-independent proteasome substrates 11*.* The ubiquitin-independent nature of ODC degradation is conserved from yeast to humans 12*.*

ODC is a rate-limiting enzyme in the biosynthesis of essential cellular polycations called polyamines. Spermidine and spermine are the most important polyamines, which, in fungi and animals, derive from ornithine 13. Polyamines are ubiquitous molecules involved in a variety of cellular functions ranging from DNA stabilization, regulation of gene expression and protein synthesis, to ion channel function and cell cycle progression 14. Increased ODC activity and elevated polyamine content have been implicated in several cancers 15,16,17. Recent phase II clinical trials have shown that the ODC inhibitor difluoromethylornithine (DFMO) reduced prostate polyamine levels in patients at risk for invasive prostate cancer 18*.* Another phase III clinical trial of a combinatorial DFMO and sulindac (a non-steroidal anti-inflammatory drug) therapy for colorectal cancer showed a significant interaction between dietary polyamines and the treatment 19*.* The biosynthesis of polyamines in yeast begins with the decarboxylation of ornithine to putrescine by ODC (Spe1). Putrescine is then converted to spermidine by spermidine synthase (Spe3), and spermidine to spermine by spermine synthase (Spe4).

Cellular polyamine levels are tightly controlled by a homeostatic control of the enzymes involved in their synthesis or catabolism 13,20,21*.* ODC levels are mainly regulated at the post-translational level in a mechanism that involves ODC antizyme (OAZ). Antizyme forms a heterodimer with ODC and targets it for ubiquitin-independent proteasomal degradation 22*.* In yeast, the binding of antizyme to ODC is necessary for the exposure of its N-terminal degron called ODS (ODC Degradation Signal) which is essential for its degradation 23. OAZ levels in the cells are in turn regulated by polyamines. *OAZ* mRNA is unusual as it has a stop codon after about one third of its coding sequence. For synthesis of full-length OAZ, the ribosome undergoes a +1 frameshift at this internal stop codon, a process that is conserved from yeast to humans 20,21. We have recently shown that polyamine binding to the nascent yeast Oaz1 polypeptide prevents a pile up of ribosomes on the *OAZ* mRNA thereby promoting the completion of Oaz1 synthesis 24. Aside from this translational regulation, polyamines also control Oaz1 degradation. Oaz1 in yeast undergoes ubiquitin-dependent degradation by the proteasome, which is inhibited by high cellular polyamine concentrations 21*.* Together, these two mechanisms lead to an increase of OAZ levels when intracellular polyamine concentrations are high, which in turn promotes ubiquitin-independent degradation of ODC.

Here we report that polyamines, in addition to the indirect enhancement of ODC degradation in *S. cerevisiae* by up-regulation of antizyme levels described above, promote ODC degradation by a third mechanism. Both *in vivo* and *in vitro* experiments show that polyamines directly stimulate antizyme-dependent ODC degradation by the proteasome. These findings identify a novel mechanism, by which a small organic metabolite controls ubiquitin-independent degradation of an enzyme involved in its synthesis.

## RESULTS

### A stable mutant of yeast antizyme

**Figure 1:**
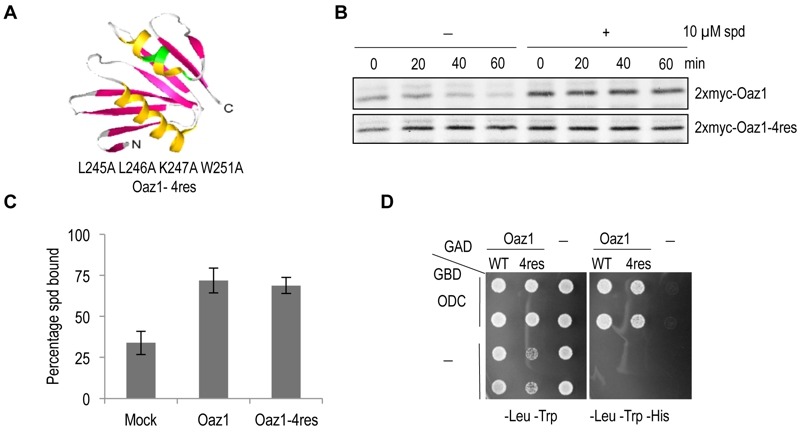
FIGURE 1: The antizyme mutant Oaz1-4res is metabolically stable in yeast cells. **(A)** Ribbon diagram of the NMR structure of rat antizyme [PDBcode 1ZO0] with β-sheets shown in pink and α-helices in yellow. Four amino acid changes (L245A L246A K247A W251A) were introduced into the yeast antizyme homologue at corresponding positions (indicated in green). The diagram was prepared using 3D molecular viewer. **(B)** Pulse-chase analysis of ^35^S-radiolabelled Oaz1 (upper panel) or Oaz1-4res (lower panel) in cells grown in the presence or absence of 10 µM spermidine (spd) showing that the stability of Oaz1-4res is independent of polyamine addition. **(C)** Spermidine binding assay showing the retention of [^3^H]-spermidine by 6His-Oaz1 or 6His-Oaz1-4res. Error bars, s.d.; *n* = 3. **(D)** Yeast two-hybrid analysis showing the interaction of Gal4 DNA binding domain (GBD) fused to ODC (GBD-ODC) with Gal4 transcription activation domain (GAD) fused to either Oaz1 or Oaz1-4res (GAD-Oaz1 or GAD-Oaz1-4res). Interaction of the two separated Gal4 domains via the polypeptides fused to them leads to reconstitution of the Gal4 transcriptional activator which controls expression of the *HIS3* gene in the reporter strain used. Interaction-mediated functional reconstitution of Gal4 can be monitored as growth on medium lacking histidine in this strain.

A mutant of antizyme with four amino acid replacements (Oaz1-4res) was generated in a targeted mutagenesis approach initially aimed to identify polyamine binding sites in antizyme. Note that in frame versions of the *OAZ1 *gene 21 were used in all experiments to avoid any effects of polyamines on translational decoding of the mRNA. Selected residues in an α helix near the C-terminal end of antizyme were mutated to alanines (Fig. 1A). Pulse chase analyses showed that Oaz1-4res was a stable protein irrespective of the presence or absence of polyamines in the growth media, whereas degradation of wild-type Oaz1 was inhibited by polyamines as reported previously (Fig. 1B) 21. We next asked whether the stable mutant Oaz1 protein binds polyamines. Oaz1-4res bound [^3^H]-spermidine with a similar efficiency as its wild-type counterpart (Fig. 1C). Yeast two-hybrid analysis showed that Oaz1-4res also retained its ability to bind ODC although a slight reduction in binding was observed (Fig. 1D). It should be noted, however, that the reporter readout in this assay (histidine prototrophic growth) does not necessarily reflect strength of interaction.

### *In vitro* characterization of ubiquitin-independent degradation of ODC

**Figure 2:**
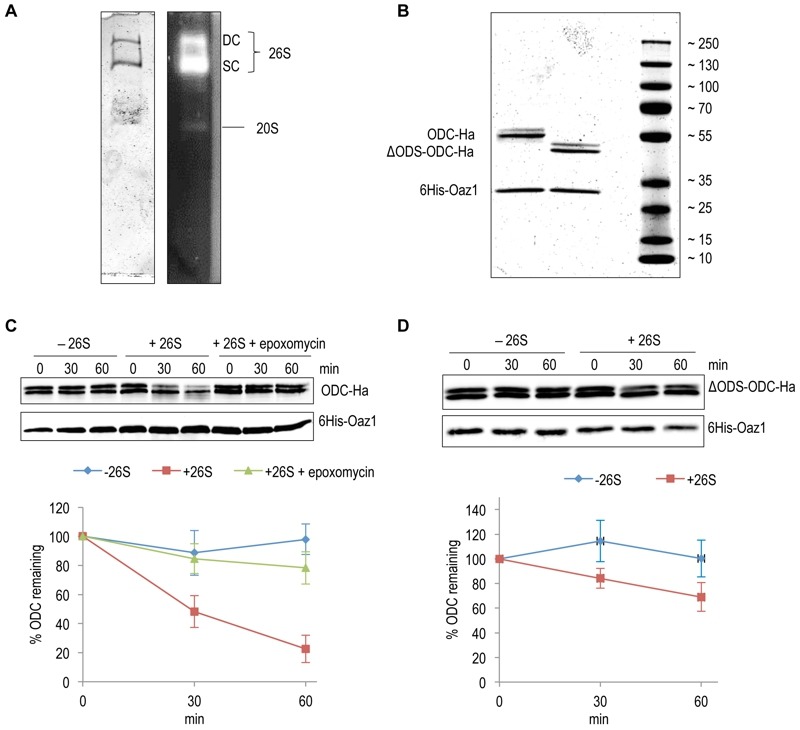
FIGURE 2: *In vitro* characterization of antizyme-mediated and ODS-dependent degradation of ODC. **(A)** Flag-tagged proteasome affinity-purified from yeast cells was analyzed by native-PAGE and Coomassie staining (left) or activity staining by overlay with the fluorogenic peptide Suc-LLVY-AMC (right). Proteasomes in this preparation were either doubly-capped (DC) with RPs on both sides of the core particle (CP), or singly-capped (SC) with only one RP attached to the CP. 20S CPs without any RPs attached to them were also present in the preparation. **(B)** SDS-PAGE analysis and Coomassie staining of 6His-Oaz1 co-purified from *E. coli* cells as heterodimer either with full length ODC (ODC-2xHa) or with N-terminally truncated ODC lacking the first 47 residues (ΔODS-ODC-2xHa). **(C)** *In vitro* degradation assays with purified 26S proteasomes and purified ODC-Oaz1 heterodimer as a substrate showing the degradation of ODC over time. 50 ng of ODC-Oaz1 heterodimer and 3 µg of 26S were mixed in 15 µL resulting in starting concentrations, respectively, of 40 nM and 80 nM. As controls, otherwise identical samples were assayed without 26S proteasome (-26S), or with the proteasome inhibitor epoxomycin. Two bands (full-length ODC-2xHa and a truncated derivative) were detected with the anti-Ha antibody, and both forms showed similar turnover rates. Therefore both bands were quantified together. In the resulting graph, values for the 0 time points were set to 100%. Error bars, s.d.; *n* = 3. **(D)** Experiments were performed as described for Fig. 2B, except that ΔODS-ODC-2xHa was used instead of full length ODC.

Using an *in vitro* assay with mouse ODC and purified 26S proteasome, Hoyt *et al.* have reproduced key *in vivo* features of ubiquitin-independent ODC degradation 25. Moreover, another *in vitro* study using yeast ODC and Oaz1 has shown that antizyme promotes ODC degradation in a ubiquitin-independent and ATP-dependent manner 26*.* Subsequent *in vivo* experiments revealed that binding of ODC monomers to antizyme is required to expose an N-terminal degron of yeast ODC called ODS (ODC Degradation Signal) 23*. *In the present study, we reconstituted ODS-dependent degradation of ODC *in vitro*. 26S proteasomes were affinity-purified using anti-Flag beads from a yeast strain with a Flag His_6_ tagged β4/Pre1 subunit. Native-PAGE analysis showed that these preparations mainly yielded active forms of the proteasome in its singly (SC) or doubly capped (DC) form, i.e. CP with one or two RPs 27 (Fig. 2A). ODC-2xHa or ΔODS-ODC-2xHa were co-purified from *Escherichia coli* cells as heterodimers together with 6His-Oaz1, which was pulled down with Ni-NTA beads binding to its 6xHis tag. The purified proteins were first characterized by SDS-PAGE and Coomassie staining. ODC-2xHa co-purified with 6His-Oaz1 as a double band (Fig. 2B)*. *The different ODC-Oaz1 heterodimers were mixed with 26S proteasomes in a buffer supplemented with ATP, and incubated at 30°C for specific time periods followed by SDS-PAGE analysis. As expected, ODC was degraded over time whereas antizyme remained stable (Fig. 2C; lanes 4-6). In the control without 26S proteasomes, in contrast, ODC was not degraded (Fig. 2C; lanes 1-3). Around 75% inhibition of degradation was observed upon addition of epoxomicin, a selective proteasome inhibitor 28 (Fig. 2C; lanes 7-9). In a similar experiment using the ΔODS variant of ODC, only around 30% degradation was observed compared to the 80% degradation observed for the full-length ODC (Fig. 2D). These results show that ODS is critical for efficient degradation of ODC in line with the *in vivo* data reported earlier 23.

### Polyamines directly enhance ODC degradation by the proteasome in yeast cells

**Figure 3:**
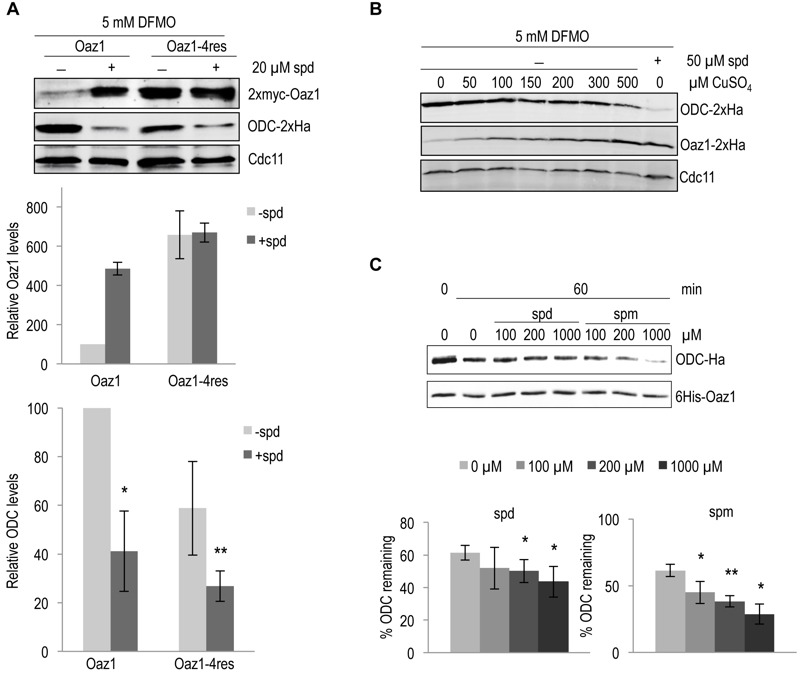
FIGURE 3: Degradation of ODC by the proteasome is directly enhanced upon polyamine addition. **(A)** *S. cerevisiae spe1-*∆* oaz1-*∆ cells were transformed with plasmids encoding ODC-2xHa and either wild-type 2xMyc-Oaz1(wt) or its 4res mutant variant. The latter proteins were encoded by in frame versions of the gene (*OAZ1-if*) that did not require ribosomal frameshifting during decoding. Western blot analysis of the steady state levels of ODC (middle panel) in the presence of either Oaz1 or Oaz1-4res (top panel) from yeast cells grown in the presence of 5 mM DFMO. 20 µM spermidine (spd) was added as indicated. Also shown is the quantification of the 2xmyc-Oaz1 (wild-type and 4res) and ODC-2xHa signals normalized to Cdc11 levels. Levels are given relative to, respectively, Oaz1 and ODC levels obtained with cells expressing wild-type Oaz1 in the absence of spd, which were set to 100%. Error bars, s.d.; *n* = 2. Significance values were calculated by paired T test comparing ODC levels to those with wild-type Oaz1 and without spd; P ≤ 0.05 (*) and P ≤ 0.01 (**). **(B)** Western blot analysis of the steady state levels of ODC-2xHa and Oaz1-2xHa in a *spe1-*Δ* oaz1-*∆ strain in the presence of 5 mM DFMO. The plasmid-encoded ODC gene (*SPE1*) was expressed from its own promoter, whereas *OAZ1-if*(wt) was expressed from the copper-inducible P*_CUP1_* promoter. CuSO_4_ was added to the medium as indicated. The last lane shows ODC and Oaz1 levels in cells grown without CuSO_4_ but with 50 µM spd showing decreased ODC levels compared to other lanes in spite of similar Oaz1 levels. **(C)** *In vitro* degradation of ODC was assayed with 0.06 µg of 26S proteasome (1.6 nM) and 100 ng of ODC-Oaz1 heterodimer (80 nM) in a volume of 15 µL and varying concentrations of either spd or spermine (spm) as indicated. The graph shows the quantification of ODC-2xHa signals. Error bars, s.d.; *n* = 3. Signficance values were calculated by paired T test comparing ODC levels to the samples incubated for 60 min without spd; P ≤ 0.05 (*) and P ≤ 0.01 (**).

Polyamines regulate cellular ODC levels by two known mechanisms, namely by inducing decoding of antizyme mRNA, and by inhibiting ubiquitin-dependent antizyme degradation 21,24*.* Here, we explored a third possible mechanism of ODC regulation. We investigated whether polyamines would directly influence antizyme-dependent ODC degradation. To address this *in vivo*, we employed yeast cells expressing the stable antizyme mutant (Oaz1-4res) described above. Important for this approach was that Oaz1-4res levels were not altered by polyamine addition (Fig. 1). Steady-state levels of ODC were determined in a strain expressing either wild-type Oaz1 or Oaz1-4res and grown in the presence of difluromethylornithine (DFMO) with or without additional spermidine. DFMO, an inhibitor of ODC and anti-cancer drug, was used to minimize the internal cellular polyamine levels 29*.* As observed previously, ODC levels dropped upon spermidine addition in cells with wild-type Oaz1, which went along with elevated Oaz1 levels (Fig. 3A, lanes 1-2) 21*.* Interestingly, however, ODC levels were also lowered further upon spermidine addition in cells with Oaz1-4res in spite of unchanged levels of the latter protein (Fig. 3A, lanes 3-4). Both versions of Oaz1 were expressed from in frame (if) variants of the *OAZ1 *gene that are not subject to polyamine regulation of decoding involving a ribosomal frameshift event 24. These data indicated that polyamines directly promote antizyme-mediated ODC degradation.

Next, we wanted to verify the conclusion derived from the experiments performed with the Oaz1-4res mutant with structurally wild-type Oaz1. This was important to exclude any influence of the four mutations present in Oaz1-4res on proteasomal targeting of ODC. To this end, ODC degradation was studied in a strain with a copper-inducible, P*_CUP1_*promoter-driven in frame (if), but otherwise wild-type version of the antizyme gene (*OAZ1-wt*). With increasing copper concentrations, Oaz1 levels increased and ODC levels declined confirming that ODC levels are inversely correlated with those of Oaz1 (Fig. 3B). Addition of spermidine caused markedly increased Oaz1 levels due to the inhibition of its ubiquitin-dependent degradation 21. Importantly for this analysis, the steady state levels of Oaz1 after full copper induction (500 µM) in

the absence of added spermidine were similar to the levels in the absence of copper induction after addition of 50 µM spermidine. In other words, now that similar Oaz1 levels were established in the absence or presence of spermidine by altering the transcriptional induction of *OAZ1 *gene expression with copper, direct effects of the polyamine on ODC degradation by the proteasome could be investigated. Indeed, the presence of spermidine caused a remarkable decrease in ODC levels in spite of similar Oaz1 levels established under these conditions (Fig. 3B; compare lanes 7 and 8). These data thus confirmed, now with a structurally wild-type Oaz1 (Fig. 3B), that spermidine exerts a direct effect on ODC targeting to the proteasome as observed before with the Oaz1-4res mutant protein (Fig. 3A).

### Polyamines directly enhance ODC degradation by the proteasome *in vitro*

To further validate our *in vivo* data, we used the *in* *vitro* ODC degradation system described above to study the direct effects of polyamines on ODC degradation. Consistent with the* in vivo *results, increased degradation of ODC was observed with increasing concentrations of either spermidine or spermine (Fig. 3C). Spermine showed a greater effect on ODC degradation than spermidine. This finding is compatible with the higher binding affinity of Oaz1 observed for spermine compared to spermidine 24.

**Figure 4:**
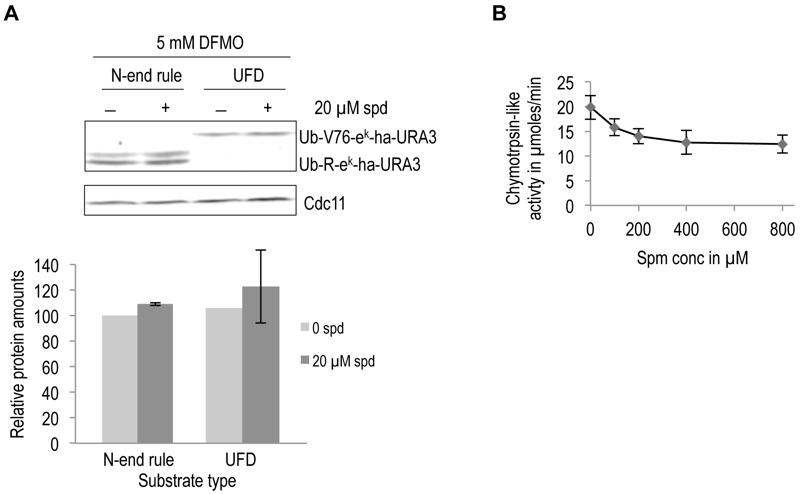
FIGURE 4: Polyamines do not enhance the degradation of ubiquitin-dependent substrates by the proteasome. **(A)** Western blot analysis of steady state levels of two ubiquitin-dependent substrates, namely, a substrate of the N-end rule pathway (Ub-R-e^k^-Ha-URA3) and a substrate of the UFD pathway (Ub-V76- e^k^-Ha-URA3). Experiments were done as described for Fig. 3A. Ha signals were quantified, normalized to the Cdc11 loading controls, and given relative to the level of protein without spermidine, which was set to 100%. Error bars, s.d.; *n* = 2. **(B)** Assay of chymotrypsin-like activity with purified proteasome (same material as shown in Fig. 2A) in the presence of increasing spermine (spm) concentrations. Error bars, s.d.; *n* = 4.

To investigate the specificity of the observed effect of polyamines on ODC degradation, we asked if polyamines have any general effect on proteolytic degradation. To address this possibility *in vivo*, we used two well characterized ubiquitin-dependent substrates, an N-end rule substrate (Ub-R-e^K^-Ha-Ura3) and a Ubiquitin Fusion Degradation (UFD) pathway substrate (Ub-V76-e^K^-Ha-Ura3) 30,31. No significant effect on degradation of these two substrates was observed upon polyamine addition to polyamine-depleted cells (Fig. 4A)*. *Additionally, the chymotrypsin-like activity of purified 26S proteasome was measured in the presence of increasing spermine concentration. A small reduction in proteasome activity was observed with polyamine addition (Fig. 4B). Taken together, the results presented above demonstrate that polyamines directly and specifically enhance ODC degradation by the proteasome.

### Polyamines do not alter the affinity of antizyme for ODC

**Figure 5:**
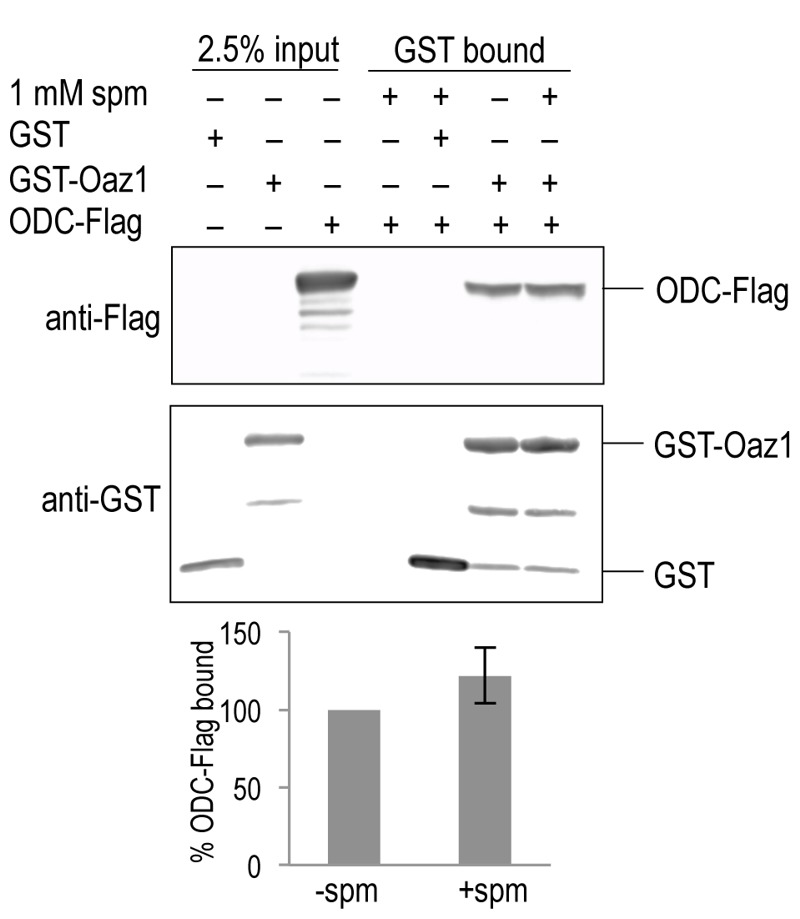
FIGURE 5: Spermine does not affect the affinity of antizyme to ODC. Co-pull down of Oaz1 and ODC in the presence or absence of 1 mM spermine (spm). Extracts from *E. coli* cells expressing the indicated tagged proteins were subjected to GST-pull down and subsequent quantitative anti-Flag western blotting for ODC-Flag detection and anti-GST for GST-Oaz1 detection. ODC-Flag signals after elution were normalized to GST-Oaz1 signals providing ODC-Flag values obtained in the absence of spm, the mean of which was set to 100%. Values obtained in the presence of spm are given in % of those obtained in its absence. Error bars, s.d.; *n* = 3.

To understand the mechanism behind the effect of polyamines on ODC degradation, we tested if polyamines changed the affinity of the ODC-antizyme interaction. To address this question, we performed co-pull down assays using epitope-tagged variants of ODC and Oaz1 expressed in *E. coli*. GST-Oaz1 bound beads were exposed to *E. coli* cell extracts overexpressing ODC-Flag in the presence or absence of spermine. Western blot analysis after GST pull down showed no significant difference in ODC-Flag binding between the samples with and without spermine (Fig. 5). This data suggested that polyamines promote ODC degradation without altering ODC-antizyme heterodimer interactions.

### Both spermidine and spermine promote antizyme and ODC degradation *in vivo *

**Figure 6:**
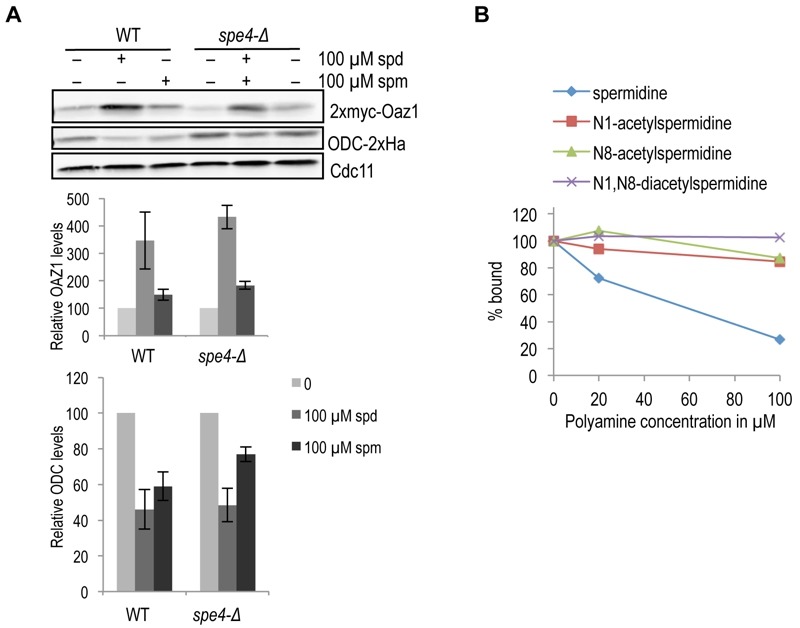
FIGURE 6: Role of polyamine subtypes and their modification in the targeting of ODC. **(A)** Western blot analysis comparing steady state levels of Oaz1 and ODC in either the wild type or a strain lacking spermine synthase (*spe4*Δ), grown with or without polyamine supplementation as indicated. Cells were transformed with plasmids expressing 2xmyc-Oaz1 from an in frame version of the *OAZ1 *gene, and with a plasmid encoding ODC-2xHa. The graph shows the results of a quantification of myc (upper part) and Ha signals (lower part) normalized to the Cdc11 loading control. Levels are given relative to the respective levels of the same proteins in cells grown without polyamine addition, which was set to 100%. Error bars, s.d.; *n* = 3. **(B)** Acetylation of spermidine inhibits its binding to antizyme. *In vitro* binding assay showing the competition between [^3^H]-spermidine and different species of acetylated spermidine for binding to 6xHis-tagged antizyme purified from *E. coli.*

As spermine binds antizyme better than spermidine 24 and also shows a greater effect on the enhancement of ODC degradation *in vitro*, we asked whether spermine is the major mediator of ODC regulation in yeast cells. We, therefore, compared the effect of spermidine and spermine on antizyme stabilization and ODC degradation in wild-type and *spe4-*Δ strains. *SPE4* encodes spermine synthase, an enzyme that mediates the conversion of spermidine to spermine. Hence, *spe4-*Δ cells are devoid of spermine 32. Antizyme degradation was similarly inhibited in both WT and *spe4-*Δ cells upon addition of spermidine or spermine (Fig. 6A, top panel). When compared to spermine, addition of spermidine had a stronger effect on the inhibition of antizyme degradation in both strains. Similarly, spermidine had a stronger (stimulatory) effect on ODC degradation than spermine (Fig. 6A, middle panel). Since we used cells expressing *OAZ1-if *constructs, these assays only monitored the effects of polyamines on Oaz1 protein stability and its capacity to mediate ODC degradation by the proteasome. The results suggest that both spermidine and spermine are capable of mediating ODC regulation in yeast cells. The relatively weaker effect of spermine on ODC targeting *in vivo* contrasts with its relatively stronger effect *in vitro* and is likely due to a lower uptake efficiency of spermine by yeast cells 33,34.

In mammals, when cellular polyamine levels are high, they are acetylated leading to their breakdown or export from the cells 35*.* High cellular polyamine levels, in addition, lead to antizyme synthesis and hence the down-regulation of ODC. We therefore asked whether acetylated polyamines might be responsible for down-regulating ODC by binding to antizyme. We performed a competition assay with [^3^H]-spermidine and various acetylated spermidine derivatives for Oaz1 binding. In this assay, mono-acetylated spermidine derivatives showed a clearly reduced binding to antizyme when compared to unmodified spermidine, and di-acetylspermidine showed no competition at all (Fig. 6B). These findings suggest that acetylated polyamines do not participate in the feedback regulation of ODC.

## DISCUSSION

### Polyamines enhance degradation of ODC

We report the identification of an additional mechanism by which polyamines influence the post-translational control of ODC, which adds a new dimension to the polyamine-mediated feedback regulation of ODC. The canonical regulation of ODC levels by polyamines takes place via up-regulation of antizyme by promoting translational decoding of its mRNA, which involves a ribosomal frameshifting event 12,20. We have previously shown that antizyme binds directly to polyamines and that co-translational binding of the nascent yeast antizyme (Oaz1) to polyamines promotes completion of its synthesis 24. Polyamines, in addition, inhibit the ubiquitin-dependent degradation of Oaz1 21*.* Together, these findings established that polyamine binding leads to an increase in the levels of Oaz1 by promoting its synthesis and stability 12*.* Higher Oaz1 levels, in turn, mediate a more rapid turnover of ODC. In the present work, we show that polyamines, both* in vivo* and *in vitro*, moreover have a direct effect on ODC by stimulating its antizyme-mediated and ubiquitin-independent degradation by the proteasome.

The difficulty in demonstrating this novel direct effect of polyamines on ODC turnover* in vivo *was to separate this mechanism from the role of polyamines in regulating the cellular levels of Oaz1. The effect of polyamines on decoding of the *OAZ1* mRNA could be eliminated by employing constructs lacking the ribosomal frameshifting site (Figure 3A and B). The identification of the metabolically stable Oaz1-4res mutant provided us with an additional new tool to investigate a direct effect of polyamines on ODC degradation. The Oaz1-4res mutant retains the ability to bind polyamines as well as to interact with ODC and target it for ubiquitin-independent degradation, but the stability of Oaz1-4res itself is not influenced by polyamines (Fig. 1). In yeast cells, wherein the internal polyamine levels were reduced by the ODC inhibitor DFMO, Oaz1-4res promoted ODC degradation more efficiently upon polyamine addition even though the levels of Oaz1-4res remained unaffected indicating that polyamine directly influence Oaz1-mediated degradation of ODC by the proteasome (Fig. 3A). Importantly, we could confirm this conclusion with an additional experimental approach that employed a structurally wild-type Oaz1, stability of which is influenced by polyamines. This could be achieved by appropriately adjusting Oaz1 synthesis using a copper-regulated promoter (Fig. 3B). Both experimental *in vivo *assays indicated that direct stimulation of Oaz1-mediated ODC degradation plays a critical role in efficiently lowering ODC levels when polyamine concentrations go up. This conclusion could be derived from the observation that even high levels of Oaz1 protein were not reducing ODC levels to the same extent when polyamine concentrations were low as when they were high (Figs. 3A and 3B). Together, these findings therefore suggest that the direct stimulation of ODC degradation by polyamines is a physiologically significant part in the negative feedback regulation of ODC in addition to the positive effect of polyamines on Oaz1 synthesis and stability. *In vitro* assays with purified 26S proteasome and ODC-antizyme heterodimer provided independent evidence supporting the direct effect of polyamines on ODC degradation observed *in vivo*. The *in vitro* effect, however, seemed smaller than expected from the *in vivo* results. A possible explanation is that the experimental *in vitro* conditions might not precisely reflect the biochemical milieu and environment in which this process occurs *in vivo*.

In the *in vitro* experiments, spermine showed a greater stimulatory effect on ODC degradation than spermidine (Fig. 3). This effect can be related to a higher binding affinity of spermine to antizyme in comparison to spermidine 24*.* The stronger effect of spermine on ODC targeting compared to spermidine *in vitro,* contrasts with its relatively weaker effect *in vivo. *The weaker effect of spermine added to the yeast culture medium is probably due to a lower uptake efficiency for this polyamine compared to spermidine 33,34. Aside from the difference in the uptake efficiencies for the different polyamines, another difficulty in comparing the magnitude of the effects observed *in vitro* and *in vivo* is the absence of solid data on the intracellular concentration of free forms of these polyamines. Nonetheless, a clear and specific stimulatory effect of polyamines on ODC degradation by the proteasome was observed both* in vivo* and *in vitro*.

At this point, the exact mechanism by which polyamines directly enhance ODC degradation remains unclear. We tested whether polyamines enhance proteasome activity or the degradation of ubiquitin-dependent substrates. Polyamines slightly inhibited chymotrypsin-like activity of the proteasome *in vitro*, and had no effect on the degradation of ubiquitin-dependent substrates in DFMO-treated yeast cells (Fig. 4). Because they bind antizyme, polyamines might enhance ODC-antizyme heterodimer formation. In pull down experiments, however, polyamines did not have any detectable effect on the binding of ODC to Oaz1 (Fig. 5). It remains a possibility that this binding assay is not sensitive enough to capture physiologically relevant small differences in binding affinity. Another possibility is that polyamine binding to the ODC-Oaz1 complex promotes ODC degradation by the proteasome without altering binding affinity between ODC and Oaz1. Two scenarios could be envisioned. Binding of polyamines to the complex might either cause a conformational change in ODC resulting in a better exposure and binding to the proteasome of its N-terminal unstructured domain 23*.* Another possibility is that polyamine binding to Oaz1 increases its affinity to an additional binding site in the proteasome 23,36*.* Further studies including structural analyses will be required to resolve this issue.

### Role of polyamine subtypes and their modification in ODC targeting

Since spermine showed a greater effect on ODC degradation *in vitro*, we asked whether spermine might be the main factor driving ODC degradation *in vivo*. To test this, we used a strain (*spe4-*∆) lacking the enzyme spermine synthase, which is unable to convert spermidine into spermine. Since there were no significant differences in ODC or Oaz1 levels detectable between wild-type and *spe4-*∆ cells (Fig. 6B), we conclude that formation of spermine from spermidine is not critical for ODC targeting *in vivo.*

Catabolism and export of polyamines is known to be initiated by their acetylation 35*.* Since these mechanisms are relevant at high polyamine concentrations that are also known to trigger ODC down-regulation, we tested whether acetylation of polyamines had an effect on their binding to antizyme. We observed that acetylation of spermidine clearly inhibited its binding to antizyme. This is likely due to the neutralization of the positive charges on polyamines by the acetyl groups. These findings indicate that acetylation of polyamines is not enhancing their roles in promoting ODC degradation.

The findings of the present work, together with previous observations, establish that polyamines act at three levels in a negative feedback loop that controls ODC. Polyamine binding to Oaz1 stimulates its synthesis, inhibits its ubiquitin-dependent degradation, and directly promotes Oaz1-mediated degradation of ODC by the proteasome. The latter is, to the best of our knowledge, the first example wherein a small natural compound directly promotes the degradation of a protein by the proteasome.

## MATERIALS AND METHODS

### Yeast media, strains and plasmids 

Yeast rich (YPD) and synthetic (S) minimal media with 2% dextrose (SD) were prepared as described 37. Spermidine (Sigma-Aldrich), spermine (Sigma-Aldrich), DFMO (kindly provided by Dr. Patrick Woster), or CuSO_4 _were added to the media at various concentrations as indicated. Strains and plasmids used in this study are listed in tables S1 and S2, respectively, in the supplementary information.

### Analysis of protein levels, stability and interactions 

Analysis of steady state proteins levels in *S. cerevisiae* cells by SDS-PAGE and immunoblotting was performed as described 21. Proteins were detected using either anti-mouse or anti-rabbit IgG secondary antibodies coupled to near-infrared fluorophores (Rockland). The blots were scanned and the signal intensities quantified using the Odyssey Infrared Imaging System (Li-COR Biosciences). For detection of epitope tags, we used the following monoclonal antibodies: The Ha epitope was detected with 16B12 monoclonal mouse antibody (Covance), Cdc11 was detected with polyclonal rabbit antibody (Santa Cruz), and the myc epitope with 9B11 mouse monoclonal antibody. For co-immunoprecipitation, proteins were extracted from *E. coli* BL21 codon^+^ cells harboring either pDG241 (GST-Oaz1), pGEX-2TX (GST), pDG273 (ODC-Flag) or pUC19 (mock) by glass bead lysis in ice-cold lysis buffer (50 mM Na-HEPES (pH 7.5), 5 mM EDTA, 1% Triton X-100) containing ‘Complete’ Protease-Inhibitor cocktail (Roche). Total protein amounts were equilibrated between GST-Oaz1 and GST lysate using the mock lysate. 800 µg of total proteins were incubated with 100 µl of glutathione beads (GE Healthcare) at 4°C for 2 h. From this step onwards, 1 mM spermine was added to certain tubes as indicated in Fig. 5. The beads were washed two times with lysis buffer, and further incubated after the addition of ODC-Flag lysate at 4°C for 2 h. Bound proteins were eluted by incubation with 125 µl elution buffer (25 mM Glutathione (Sigma-Aldrich), 20 mM NaOH in lysis buffer) at 4°C for 90 min. The samples were then analyzed by SDS-PAGE and western blotting as described above. The GST and Flag epitopes were detected using rabbit polyclonal antibody (Santa Cruz) and mouse monoclonal M2 antibody (Sigma-Aldrich), respectively. Pulse chase analysis 38 and yeast two-hybrid assays 39 were carried out as described earlier.

### Protein purification

26S proteasomes were purified as described previously 40 from yeast strain MO24, in which the *PRE1* gene, encoding the 20S core particle subunit Pre1, has been stably modified to express a C-terminally Flag-6His tagged version. The purity and activity of the proteasomes were analyzed by Native-PAGE 27 followed by either Coomassie staining or in-gel chymotrypsin-like activity degradation assay 27*.* 6His-OAZ1 and 6His-OAZ1-4res proteins were affinity-purified from *E. coli* strain Rosetta (Merck) transformed with pDG240 (6His-Oaz1) or pDG246 (6His-Oaz1-4res) respectively, as described earlier 24*.* 6His-Oaz1 and ODC-2xHa or 6His-Oaz1-ΔODS-ODC-2xHa were co-expressed in *E. coli* strain Rosetta and Ni-affinity purified as described above with a few variations. The lysis buffer used was buffer B (25 mM Na-HEPES, pH 7.8, 5 mM MgCl_2_, 25 mM KCl, 10% glycerol). After elution of the protein, imidazole was removed using NAP^TM^-5 (GE Healthcare) columns. The purity of the eluted proteins was evaluated by SDS-PAGE followed by Coomassie staining as described above.

### Proteasomal peptidase activity assay 

The chymotrypsin-like activity was measured using Suc-LLVY-AMC as a substrate as described earlier 41 with the following modifications. The reactions were carried out using 0.06 µg of purified 26S proteasome and varying amounts of spermine (as shown in Fig. 4B) in 90 µL of buffer B supplemented with 1 mM ATP and 1 mM DTT. 10 µL of a 1 mg/mL Suc-LLVY-AMC solution was added to this mixture.

### *In vitro* degradation assay

Degradation assays were performed as described earlier 40 with the following variations. The amounts of 26S and substrate used are as indicated in Figs. 2 and 3. The reaction buffer (buffer B) was supplemented with 1 mM ATP and 1 mM DTT. To inhibit proteasomal activity; proteasomes were pre-treated with 100 µM epoxomycin (Enzo life sciences) at 30°C for 45 min before adding to the degradation assays. Wherever indicated (refers to Fig. 3C), the reactions were supplemented with either spermidine or spermine. The degradation reactions were carried out at 30°C for various time points as indicated in Figs. 2 and 3 followed by SDS-PAGE and Western blot analysis. ODC-2xHa was detected with 16B12 mouse monoclonal antibody and 6His-Oaz1 with anti-Oaz1 polyclonal antibody.

### Polyamine binding assay

Polyamine binding was measured as described earlier 24,42*.* The [^3^H]-spermidine competition assay was performed using 10 µM of [^3^H]-spermidine and the indicated concentrations (Fig. 6B) of the following acetyl polyamines; N1-acetylspermidine (Wako), N8-acetylspermidine (Sigma-Aldrich) and N1, N8-diacetylspermidine (Wako).

## SUPPLEMENTAL MATERIAL

Click here for supplemental data file.

All supplemental data for this article are also available online at http://microbialcell.com/researcharticles/polyamines-directly-promote-antizyme-mediated-degradation-of-ornithine-decarboxylase-by-the-proteasome/.
