# Supplementary material for: Polyamines directly promote antizyme-mediated degradation of ornithine decarboxylase by the proteasome
Source: Microb Cell. 2015 May 20;2(6):197–207. doi: 10.15698/mic2015.06.206 (PMC5349141; doi:10.15698/mic2015.06.206)
Supplement: Supplementary file 1 [file mic-02-197-s01.pdf]

Supplementary Table 1: Saccharomyces cerevisiae strains used in this study

**Strain**	**Genotype**	**Source**
JD47-13C	*MATa his3Δ200 leu2-3,112 lys2-801 trp1Δ63 ura3-52*	[1]
PJ69-4A	*MATa his3Δ200 leu2-3,112 lys2-801 trp1-901 ura3-52 gal4Δ gal80Δ lys2::GAL1-HIS3 GAL2-ADE2 met2::GAL7-lacZ*	[2]
MO24	*MATa his3Δ200 leu2-3,112 lys2-801 trp1Δ63 ura3-52 pre1::PRE1-FLAG-6xHIS*	[3]
spe1-Δ oaz1-Δ	*MATα spe1Δ::KanMX4 oaz1Δ::natNT2 his3Δ1 leu2Δ0 lys2Δ0 ura3Δ0*	[4]
BY4741	*MATa his3Δ*1 *leu2Δ*0 *met15Δ*0 *ura3Δ*0	Euroscarf
spe4-Δ	*MATa his3Δ*1 *leu2Δ*0 *met15Δ*0 *ura3Δ*0 *spe4Δ::KAN*	Euroscarf

Supplementary Table 2: Plasmids used in this study

Name	Details	Source
YCplac33	CEN/URA3	[5]
YCplac111	CEN/LEU2	[5]

pPM323	P*CUP1*-2xmyc-OAZ1-if-T*CYC1*, CEN/URA3	[6]
pPM381	P*CUP1*-2xmyc-OAZ1-if $_{L245A,L246A,K247A,W251A}$ -T*CYC1*, CEN/URA3	This study
pDG240	pET11a-6HIS-OAZ1(codon optimised for *E.coli*)	[6]
pDG246	pET11a-6HIS-OAZ1$_{L245A,L246A,K247A,W251A}$ (codon optimised for *E.coli*)	This study
pGAD-C2	GAL4-AD, 2μ/LEU2	[2]
pGBD-C2	GAL4-BD, 2μ/TRP1	[2]
pPM123	GAD-OAZ1-if-2xha, 2μ/LEU2	[4]
pDG155	GBD-ODC, 2μ/TRP1	This study
pRB11	pET11a-6HIS-OAZ1(codon optimised for *E.coli*)-pQE-ODC-2xHa	This study
pRB12	pET11a-6HIS-OAZ1(codon optimised for *E.coli*)-pQE-ΔN$_{47}$-ODC-2xHa	This study
pPM97	P*ODC*-ODC-2xha-T*CYC1*, CEN/LEU2	[4]
pPM94	P*CUP1*-OAZ1-2xha-T*CYC1*, CEN/URA3	[7]
pMAF17	P*CUP1*-Ub-R-ha-eK-URA3-T*CYC1*, CEN/LEU2	Derivative of pMAF18
pMAF18	P*CUP1*-Ub-V76-ha-eK-URA3-T*CYC1*, CEN/LEU2	[8]

pGEX-4T-2	GST	GE Healthcare
pDG241	pGEX4T-2-GST-OAZ1(codon optimized for *E.coli*)	This study
pDG273	pET11a-ODC-FLAG	This study

References to supplementary tables

1. Ramos PC, Höckendorff J, Johnson ES, Varshavsky A, Dohmen RJ (**1998**). Ump1p Is Required for Proper Maturation of the 20S Proteasome and Becomes Its Substrate upon Completion of the Assembly. **Cell** 92(4): 489-499. doi: 10.1016/s0092-8674(00)80942-3

2. James P, Halladay J, Craig EA (**1996**). Genomic libraries and a host strain designed for highly efficient two-hybrid selection in yeast. **Genetics** 144: 1425-1436.

3. Kock M, Nunes MN, Hemann M, Kube S, Dohmen RJ, Herzog F, Ramos PC, Wendler P (**2015**) Proteasome assembly from 15S precursors involves major conformational changes and recycling of the Pba1–Pba2 chaperone. **Nature Commun** 22: 6123. doi: 10.1038/ncomms7123

4. Gödderz D, Schäfer E, Palanimurugan R, Dohmen RJ (**2011**). The N-terminal unstructured domain of yeast ODC functions as a transplantable and replaceable ubiquitin-independent degron. **J Mol Biol** 407: 354-367. doi: 10.1016/j.jmb.2011.01.051

5. Gietz RD, Sugino A (**1988**). New yeast-Escherichia coli shuttle vectors constructed with in vitro mutagenized yeast genes lacking six-base pair restriction sites. **Gene** 74: 527-534.

6. Kurian L, Palanimurugan R, Godderz D, Dohmen RJ (**2011**). Polyamine sensing by nascent ornithine decarboxylase antizyme stimulates decoding of its mRNA. **Nature** 477: 490-494. doi: 10.1038/nature10393

7. Palanimurugan R, Scheel H, Hofmann K, Dohmen RJ (**2004**). Polyamines regulate their synthesis by inducing expression and blocking degradation of ODC antizyme. **EMBO J** 23: 4857-4867. doi: 10.1038/sj.emboj.7600473

8. Gowda NKC, Kandasamy G, Froehlich MS, Dohmen RJ, and Andréasson C (2013) Hsp70 nucleotide exchange factor Fes1 is essential for ubiquitin-dependent degradation of misfolded cytosolic proteins. **Proc Natl Acad Sci U SA** 110: 5975-5980. doi: 10.1073/pnas.1216778110
